# Survival Comparisons Between Early Male and Female Breast Cancer Patients

**DOI:** 10.1038/s41598-018-26199-6

**Published:** 2018-06-11

**Authors:** Kang Wang, Qiu-Juan Wang, Yong-Fu Xiong, Yang Shi, Wen-Jing Yang, Xiang Zhang, Hong-Yuan Li

**Affiliations:** 1 Department of the Endocrine and Breast Surgery, The First Affiliated hospital of Chongqing Medical university, Chongqing Medical University, Chongqing, 400016 China; 2 Department of Epidemiology and Biostatistics, West China School of Public Health, Sichuan University, Sichuan, China; 3 Department of the Gastrointestinal Surgery, The First Affiliated hospital of Chongqing Medical university, Chongqing Medical University, Chongqing, 400016 China

## Abstract

We aimed to compare the overall survival (OS) and standardized mortality rate (SMR) of the male breast cancer (MBC) with female breast cancer (FBC) after propensity score matching. Based on the Surveillance, Epidemiology, and End Results (SEER), the early breast cancer patients (T_1–2_N_0–2_M_0_) were extracted from 1998–2007. This study included 1,111 and 2,151 patients with early MBC and FBC, respectively, whose clinicopathological characteristics were well balanced. At a mean follow-up time of 97 months, 10-year OS rate was 58.3% in the MBC group and 68.7% in the FBC (log-rank test, P < 0.001; hazard ratio (HR) = 1.45, 95% confidence interval (CI) = 1.29 to 1.64). Adjusted HR for OS between MBC and FBC were revealed from propensity score matched-multivariable Cox proportional hazards models (HR = 1.53, 95% CI = 1.35 to 1.73). Similar adjusted SMRs between MBC and FBC ((SMR = 1.98, 95% CI = 1.83,2.14) for FBC and (SMR = 2.07, 95% CI = 1.88–2.28) for MBC) were observed. The nomogram was constructed for FBC, and predicted probabilities were generally good (C-index = 0.71), whose area under curve is higher than TNM stage classification (0.74 vs 0.62). OS was significantly decreased among early MBC patients compared with FBC, but similar SMRs and its trends by age groups were observed between MBC and FBC except for young patients.

## Introduction

Male breast cancer (MBC) is rare, accounting for 0.11% of all male malignancies and less than 1% of breast cancer^1^, nevertheless, MBC incidence continues to rise at an annual rate of 1.1%^2^. Considering the limited numbers of studies exclusively investigating its clinicopathological characteristics and treatment strategies, current consensuses of MBC management are extrapolated from knowledge of female breast cancer (FBC)^3,4^.

Since MBC is usually diagnosed at an advanced stage^5,6^, available evidence indicates a worse prognosis compared with FBC^6–8^. Still, recent reports documented that the prevalence of early stage (stage I and II) disease has caught up with and surpassed that of stage III and IV^7,9^. Only few comparative studies^7^ with small sample size matched the clinicopathological variables, including age, primary tumor size, nodal involvement, hormone receptor status, status of human epidermal growth factor receptor 2 (HER2) and grade. Interestingly, MBC tend to be ductal with high expression of estrogen and progesterone receptors (ER and PR) shown consistently in previous case series reports^5,6,10,11^. The most common staging system for breast cancer is the Tumor/Node/Metastasis (TNM)/American Joint Committee on Cancer (AJCC) system, and the recently updated 8th AJCC staging included an integration of both anatomical and biological classification proposed by MD-Anderson in 2011 for non-metastatic breast cancer patients before surgery^12,13^. Striking disparity of prognosis between MBC and FBC suggests the need for the establishment of individual stage system for patients with MBC.

Nomograms, as reliable and convenient tools, have been widely used in clinical oncology recently^14,15^. They can quantitatively predict the prognosis of certain patients using vital prognostic factors, and illustrated the visualized results^16^. To best of our knowledge, few studies conducted matched comparisons between MBC and FBC, and successfully predicted the prognosis of early MBC using nomogram. Therefore, we have investigated the differences of prognosis between MBC and FBC, and constructed a comprehensive and practical nomogram based on the National Cancer Institute’s Surveillance, Epidemiology, and End Results (SEER) program to predict the overall survival (OS).

## Materials and Methods

### Patients selection and data processing

The SEER database (http://seer.cancer.gov/) sponsored by the National Cancer Institute covered 18 population-based registries, whose cancer data represented a large proportion (30%) of American people. The SEER*Stat software was used to extract relevant information released most recently through April 2017, including patient identification, year of diagnosis, age, race/ethnicity, histological type, nuclear grade, ER, PR, adjusted AJCC 6th TNM staging classification, surgery type, chemotherapy, radiation therapy, survival status and month. International Classification of Diseases for Oncology (ICD-O-3)^17^ was used to identify the primary cancer site and histology. We selected eligible patients with adherence of inclusion criteria as follows: (1) BC patients aged 18–79 years old was diagnosed from January 1998 to December 2007. (2) MBC was defined as early stage by TNM stage, including T_1_N_0_M_0_, T_1_N_1_M_0_, T_2_N_0_M_0_, T_2_N_1_M_0_. (3) Vital status of MBC patients was known, which was stratified into living and all-cause deaths. We also excluded men as follows: (1) BC patients were primarily diagnosed as bilateral or multiple primary tumors, and BC with distant metastasis was also excluded. (2) BC patients with relevant variables unknown, such as ethnicity, marital status, histological grade (I-III), histological type, positive lymph nodes, status of ER or/and PR, radiotherapy, chemotherapy, surgery type and survival data, were excluded after screening. Before propensity score matched with FBC, an eligible cohort involving 1,207 men and 219,992 women with early BC was merged by SEER*Stat software, and permission to access this research data was received (Reference No: 10153-Nov2016). The study was approved by The Ethics Committee of the First Affiliated Hospital of Chongqing Medical University.

### Statistical analysis

Before comparing the OS between MBC and FBC patients, we carried out propensity score matching to adjust potential confounding factors using “MatchIt” R package^18^. The year of diagnosis, age of patients, race, marital status, T and N stages based on adjusted 6^th^ AJCC, histological type, grade, ER, PR, surgery, chemotherapy and radiotherapy data were used to merged propensity scores for each individual through a logistic regression model, and balanced group have a ratio of approximately two FBC to every MBC. After propensity scored matched by above clinicopathologic variables, the whole study included MBC (n = 1,111) and FBC (n = 2,151) cohorts. We evaluated the distribution of the gender in different subgroups using Pearson Chi-squared tests.

We conducted log-rank tests and Cox proportion hazard regressions which were weighted by propensity scores to assess the differences between MBC and FBC in OS, and calculated hazard ratios (HRs) with 95% confidence interval (CI). All the adjusted HRs were acquired when fitting multivariable models, which involved covariates to adjust for aforementioned variables in propensity score matching procedure. Furthermore, subgroup analyses were conducted to assess the difference of survival by gender across potential modifiers. Additionally, to compare the relative risk of death between MBC and FBC after considering general population deaths, we calculated the standardized mortality ratio (SMR)^19^ that is defined as the ratio of observed number of deaths among patients to the expected number of deaths in the general population. The corresponding 95% CI were calculated for each SMR by assuming that the observed deaths followed a Poisson distribution^19^. Modifiers such as year of diagnosis and race was adjusted when calculating the SMRs by age at diagnosis (<45, 46–55, 56–65, 66–75, 76–85, +85). The expected number of deaths were estimated using the general population mortality rates stratified by age obtained from the Centers and Disease Control and Prevention (https://www.cdc.gov/), and the homogeneity of SMR was assessed by a likelihood ratio test.

We conducted univariate and multivariate analyses to determine potential prognostic variable on OS among MBC patients, which were used to construct the nomogram. To develop an individual prediction tool to forecast the survival of MBC, a nomogram was constructed using MBC cohort based on *rms* package in R software, which was validated internally through 200 bootstrap resamples. Concordance index (C-index) was calculated for the evaluation of the performance of predicting and discrimination ability by test concordance between predicted probability and actual outcome. Similarly, to achieve visualization, calibration of this nomogram was conducted by comparing the predicted survival with the observed survival in both, and a 45-degree diagonal line was deemed to a perfectly calibrated model. In addition, the probability of OS was predicted as a point by the nomogram or TNM stage, and we conducted receiver operating characteristic (ROC) curve to assess sensitivity and specificity of two survival prediction tools. Areas under curve (AUC) were also calculated for quantitative analyses.

All P values reported are two-sided, which less than 0.05 were considered statistically significant. All analyses were conducted using R software (version 3.2.5).

### Outcomes

#### Patient characteristics

**Figure 1:**
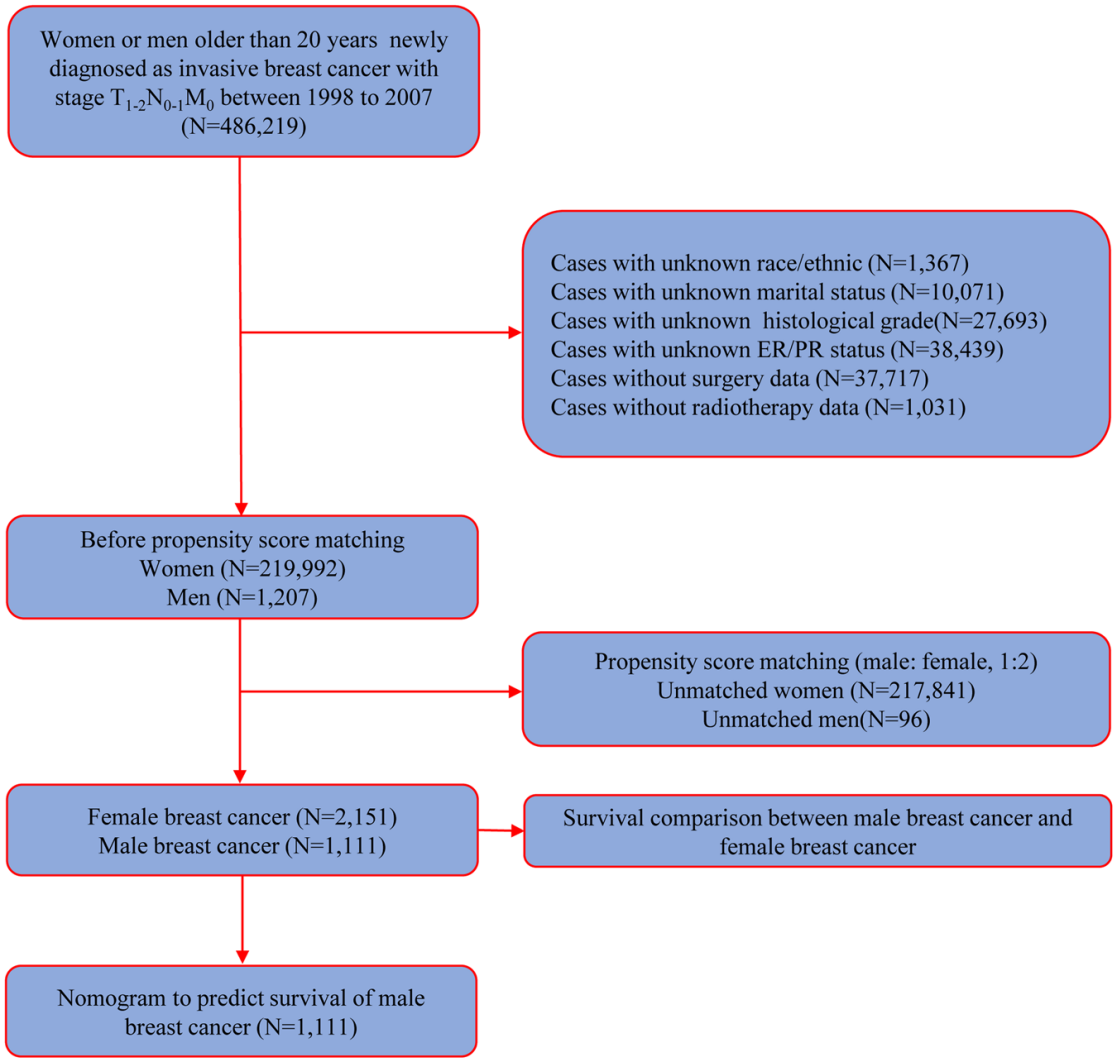
Flow chart for the SEER data screening.

**Figure 2:**
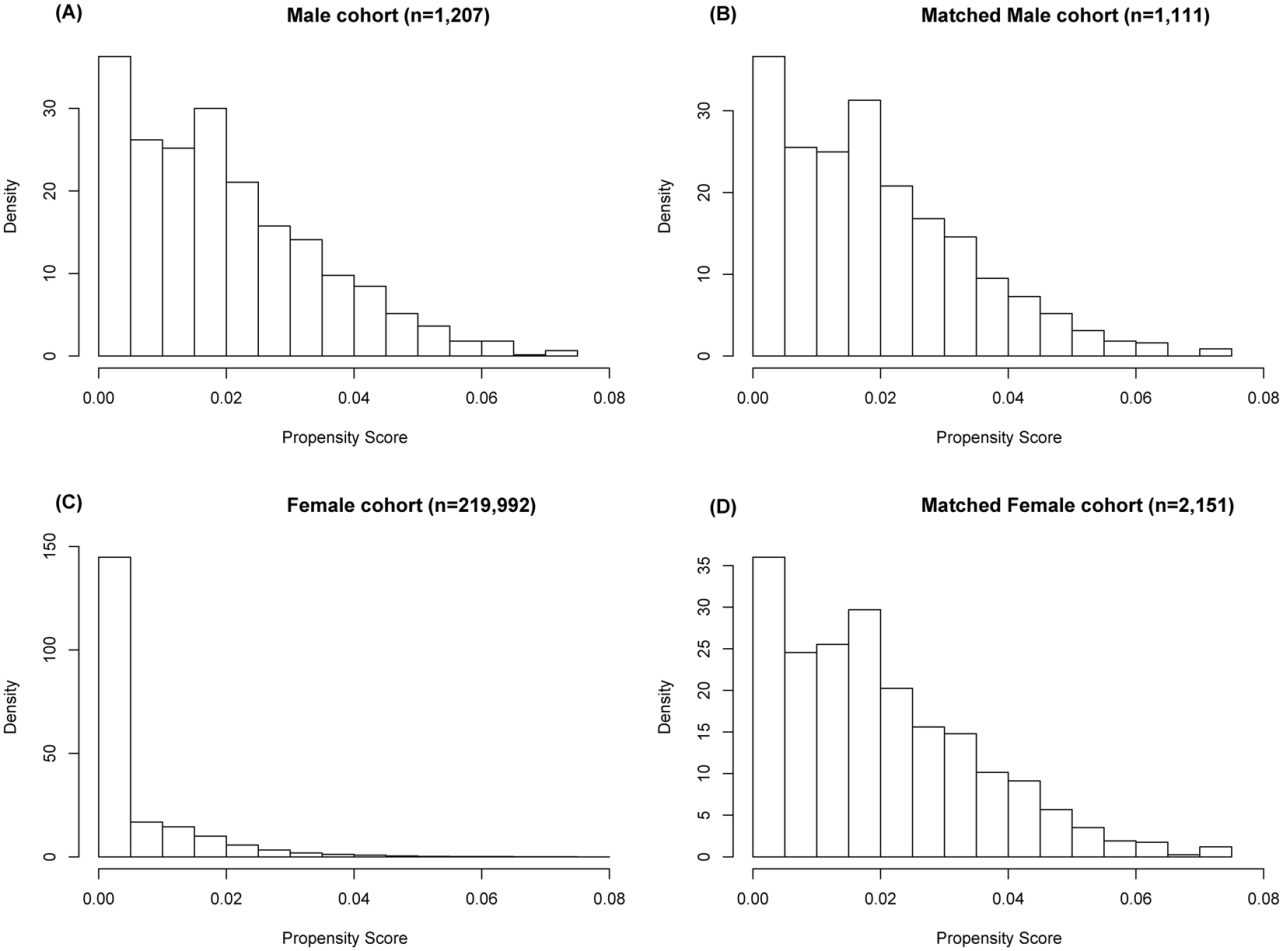
Distribution of propensity score before and after propensity score analysis. (**A**) and (**C**) show the distribution of the propensity score for patients with male and female breast cancer before the matching procedure, respectively. (**B**) and (**D**) demonstrate the distribution of the propensity score after full propensity score matching.

A total of 3,262 eligible patients were enrolled in this study on the basis of inclusion criteria. The flow chart was shown in Fig. 1. Of these patients, 2,151 BC patients were MBC, and 1,111 were FBC. Table 1 illustrated clinicopathological characteristics of all patients included, with the exception of histological type, and residual factors were balanced after considering propensity score adjustments (Fig. 2).

**Table 1:** Patients characteristics by gender after propensity score matching.

Characteristic	No. of Patients (%)		*P*
	MBC (1,111)	FBC (2,151)	
**Year of diagnosis**			
1998–2000	186 (16.7)	345 (16.0)	0.753
2001–2003	332 (29.9)	629 (29.2)	
2004–2007	593 (53.4)	1,177 (54.7)	
**Age**, **years**			
<40	25 (2.3)	40 (1.9)	0.985
40–49	116 (10.4)	225 (10.5)	
50–59	260 (23.4)	512 (23.8)	
60–69	302 (27.2)	590 (27.4)	
70–79	244 (22.0)	466 (21.7)	
>80	164 (14.8)	318 (14.8)	
**Race/ethnic**			
White	953 (85.8)	1,867 (86.8)	0.419
Black	103 (9.3)	171 (7.9)	
Other^a^	55 (5.0)	113 (5.3)	
**Marital status**			
Single	292 (26.3)	554 (25.8)	0.745
Married	819 (73.7)	1,597 (74.2)	
**AJCC T stage**			
T1mic	35 (3.2)	58 (2.7)	0.965
T1a	34 (3.1)	66 (3.1)	
T1b	104 (9.4)	206 (9.6)	
T1c	467 (42.0)	903 (42.0)	
T2	471 (42.4)	918 (42.7)	
**AJCC N stage**			
N0	661 (59.5)	1,295 (60.2)	0.881
N1	366 (32.9)	690 (32.1)	
N2	84 (7.6)	166 (7.7)	
**Histological**			
Ductal	943 (84.9)	1,536 (71.4)	<0.001
Lobular	7 (0.6)	213 (9.9)	
Other	161 (14.5)	402 (18.7)	
**Grade**			
I	171 (15.4)	316 (14.7)	0.833
II	569 (51.2)	1,121 (52.1)	
III	371 (33.4)	714 (33.2)	
**ER**			
Negative	49 (4.4)	110 (5.1)	0.377
Positive	1,062 (95.6)	2,041 (94.9)	
**PR**			
Negative	171 (15.4)	330 (15.3)	0.970
Positive	940 (84.6)	1821 (84.7)	
**Chemotherapy**			
No	684 (61.6)	1,318 (61.3)	0.871
Yes	427 (38.4)	883 (38.7)	
**Radiotherapy**			
No	846 (76.1)	1,642 (76.3)	0.904
Yes	265 (23.9)	509 (23.7)	
**Surgery**			
No	8 (0.7)	8 (0.4)	0.370
BCS	129 (11.6)	261 (12.1)	
Mastectomy	974 (87.7)	1,882 (87.5)	

^a^American Indian/AK Native, Asian/Pacific Islander.

Abbreviations: MBC, male breast cancer; FBC, female breast cancer; AJCC, American Joint Committee on Cancer system; ER, estrogen-receptor; PR, progesterone receptor; BCS, breast conserving surgery.

#### Overall survival comparison of MBC versus FBC

**Figure 3:**
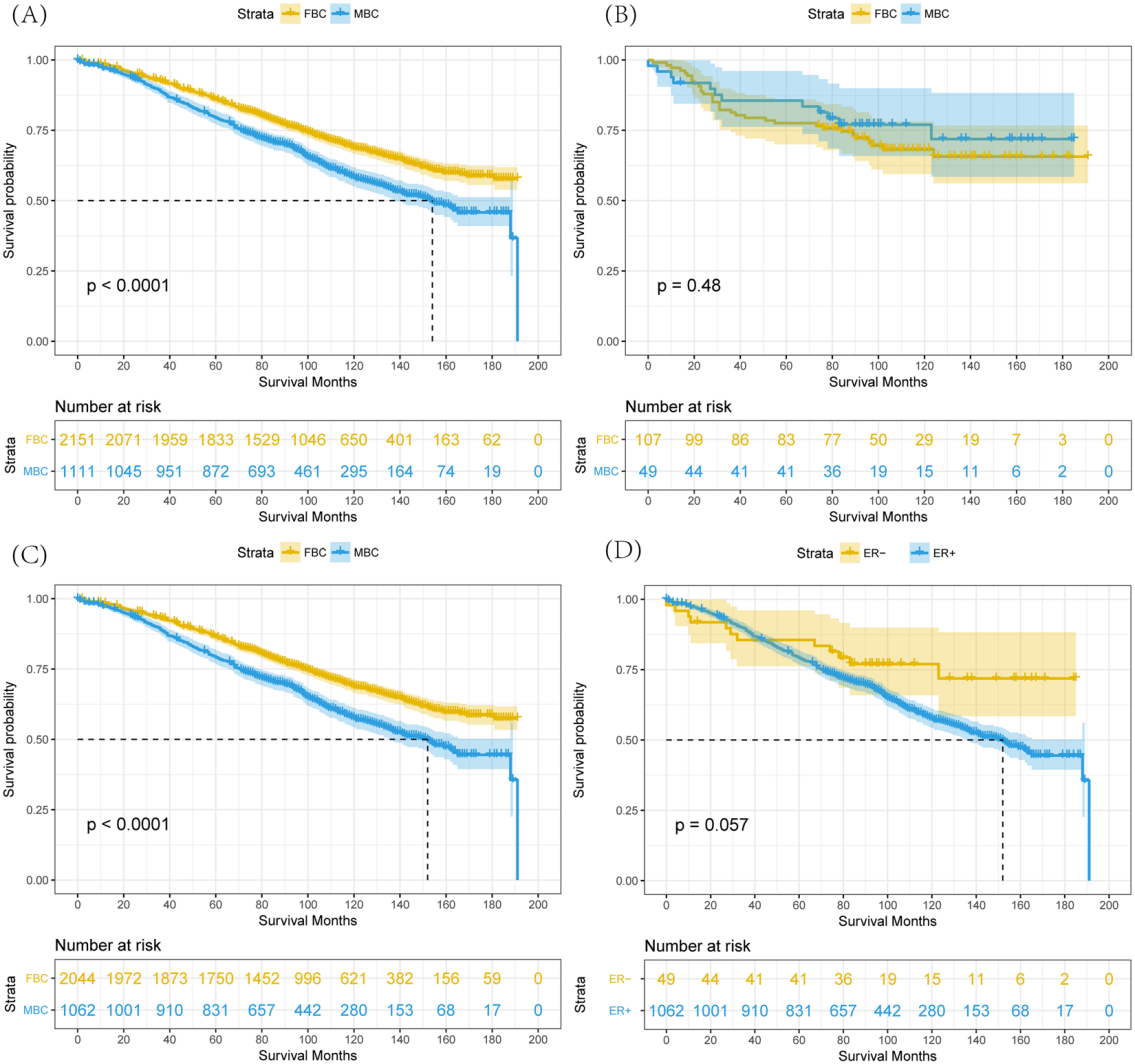
(**A**) Overall survival curves of male matched with female breast cancer patients. (**B**) Overall survival curves of ER- negative male compared with ER- negative female breast cancer patients. (**C**) Overall survival curves of ER-positive male compared with ER-positive female breast cancer patients. (**D**) Overall survival curves of ER- positive compared with ER-negative male breast cancer patients.

At a mean follow-up time of 97 months, there were 435 (13.3%) deaths and 635 (19.5%) deaths from FBC and MBC, respectively. 10-year OS rate matched by propensity score was 58.3% in the MBC group and 68.7% in the FBC (log-rank test, P < 0.001; HR = 1.45, 95% CI = 1.29 to 1.64; Fig. 3A).

**Figure 4:**
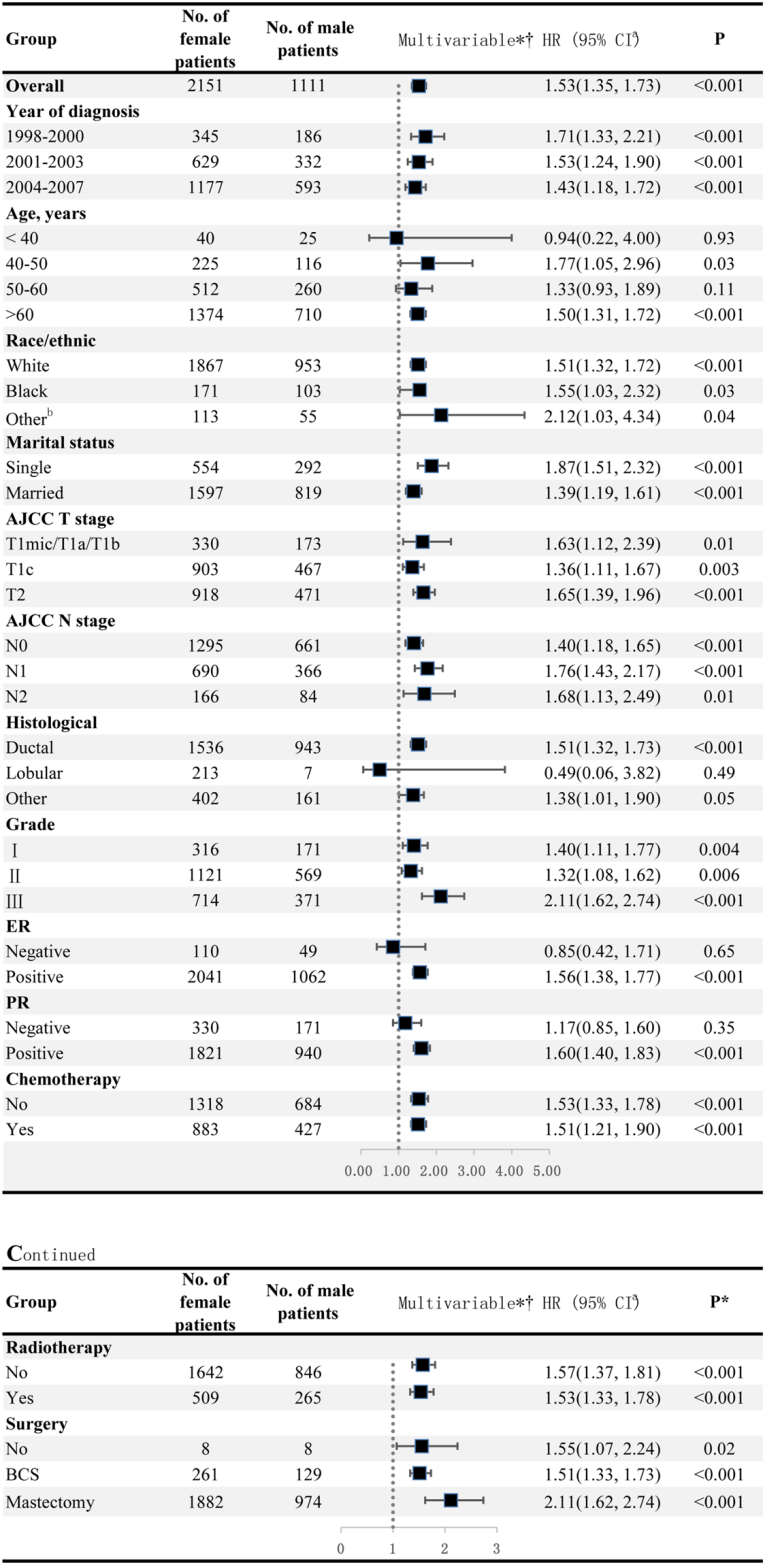
Hazard ratio comparing Overall survival between male and female breast cancer patients according to clinicopathological variables. ^*^Matched by propensity score. ^†^Multivariate analysis adjusted by year of diagnosis, age of patients, race, marital status, AJCC T stage, AJCC N stage, status of ER and PR, histological type, nuclear grade, chemotherapy, radiotherapy and surgery. ^a^The square represents the RR for each category, and horizontal line across each square represents the 95% confidence interval. The dotted line in the middle represents an invalid line. ^b^American Indian/AK Native, Asian/Pacific Islander. Abbreviations: HR, hazard risk; CI, confidence interval; ER, estrogen-receptor; PR, progesterone receptor; BCS, breast conserving surgery.

Adjusted hazard ratios for OS between MBC and FBC were revealed from inverse propensity score matched-multivariable Cox proportional hazards models with year of diagnosis, age, marital status, histologic type, nuclear grade, ER, PR, AJCC T stage, AJCC N stage, surgery, chemotherapy and radiation therapy (HR = 1.53, 95% CI = 1.35 to 1.73). Figure 4 showed the results of interaction analyses, survival advantage among FBC versus MBC was not observed in subjects with age less than 40 years, lobular or ER-negative BC. Similarly, other interaction effects on OS based on prior modifiers were also analyzed through multivariate Cox proportional hazard model (Table 2).

**Table 2:** Prognostic factors for overall survival among male breast cancer patients.

Variable	Overall Survival			
	Univariate Analysis^*^		Multivariate Analysis^†^	
	HR (95% CI)	P value	HR (95% CI)	P value
**Year of diagnosis**				
1998–2000	Reference	0.66	Reference	0.17
2001–2003	0.90 (0.69, 1.16)	0.41	0.87 (0.67, 1.13)	0.30
204–2007	0.90 (0.69, 1.16)	0.41	0.78 (0.60, 1.01)	0.06
**Age**, **years**				
<40	Reference	<0.001	Reference	<0.001
40–50	1.38 (0.53, 3.55)	0.51	1.00 (0.38, 2.61)	1.00
50–60	1.08 (0.43, 2.71)	0.87	0.90 (0.35, 2.27)	0.82
>60	3.53 (1.46, 8.53)	0.005	2.82 (1.14, 6.92)	0.02
**Race/ethnic**				
White	Reference	0.11	Reference	0.02
Black	1.25 (0.92, 1.71)	0.15	1.45 (1.05, 2.00)	0.02
Other^a^	0.70 (0.42, 1.15)	0.15	0.68 (0.41, 1.12)	0.13
**Marital status**				
Single	Reference		Reference	
Married	0.56 (0.46, 0.69)	<0.001	0.55 (0.45, 0.67)	<0.001
**AJCC T stage**				
T1mic/T1a/T1b	Reference	<0.001	Reference	<0.001
T1c	1.33 (0.95, 1.84)	0.10	1.28 (0.91, 1.79)	0.16
T2	2.42 (1.76, 3.32)	<0.001	2.09 (1.50, 2.91)	<0.001
**AJCC N stage**				
N0	Reference	<0.001	Reference	<0.001
N1	1.42 (1.16, 1.73)	0.001	1.87 (1.50, 2.32)	<0.001
N2	1.70 (1.22, 2.35)	0.002	2.60 (1.80, 3.75)	<0.001
**AJCC Stage**				
I	Reference	<0.001	\	\
IIa	1.55 (1.22, 1.97)	<0.001	\	\
IIb	2.58 (1.99, 3.35)	<0.001	\	\
IIIa	2.24 (1.59, 3.15)	<0.001	\	\
**Histological type**				
Lobular	Reference	0.45	Reference	0.40
Ductal	0.30 (0.04, 2.10)	0.22	0.31 (0.04, 2.20)	0.24
Others	0.96 (0.73, 1.26)	0.75	1.10 (0.83, 1.46)	0.53
**Grade**				
I	Reference	0.002	Reference	0.003
II	1.67 (1.22, 2.29)	0.002	1.69 (1.22, 2.35)	0.002
III	1.78 (1.28, 2.47)	0.001	1.81 (1.28, 2.55)	0.001
**ER status**				
Negative	Reference		Reference	
Positive	1.73 (0.98, 3.08)	0.06	1.51 (0.82, 2.81)	0.19
**PR status**				
Negative	Reference		Reference	
Positive	1.13 (0.86, 1.48)	0.38	1.09 (0.81, 1.47)	0.57
**Surgery**				
No surgery	Reference	0.005	Reference	0.006
BCS	0.26 (0.11, 0.60)	0.002	0.37 (0.15, 0.92)	0.03
Mastectomy	0.26 (0.12, 0.59)	0.001	0.29 (0.12, 0.68)	0.005
**Radiotherapy**				
No	Reference		Reference	
Yes	0.80 (0.64, 1.00)	0.05	0.70 (0.55, 0.91)	0.007
**Chemotherapy**				
No	Reference		Reference	
Yes	0.59 (0.48, 0.72)	<0.001	0.54 (0.43. 0.69)	<0.001

^*^Univariable Cox regression analysis.

^†^Multivariate analysis adjusted by year of diagnosis, age of patients, race, marital status, AJCC T stage, AJCC N stage, status of ER and PR, histological type, nuclear grade, chemotherapy, radiotherapy and surgery.

^a^American Indian/AK Native, Asian/Pacific Islander.

Abbreviations: HR, hazard ratio; CI, confidence intervals; MBC, male breast cancer; FBC, female breast cancer; AJCC, American Joint Committee on Cancer system; ER, estrogen-receptor; PR, progesterone receptor; BCS, breast conserving surgery.

**Figure 5:**
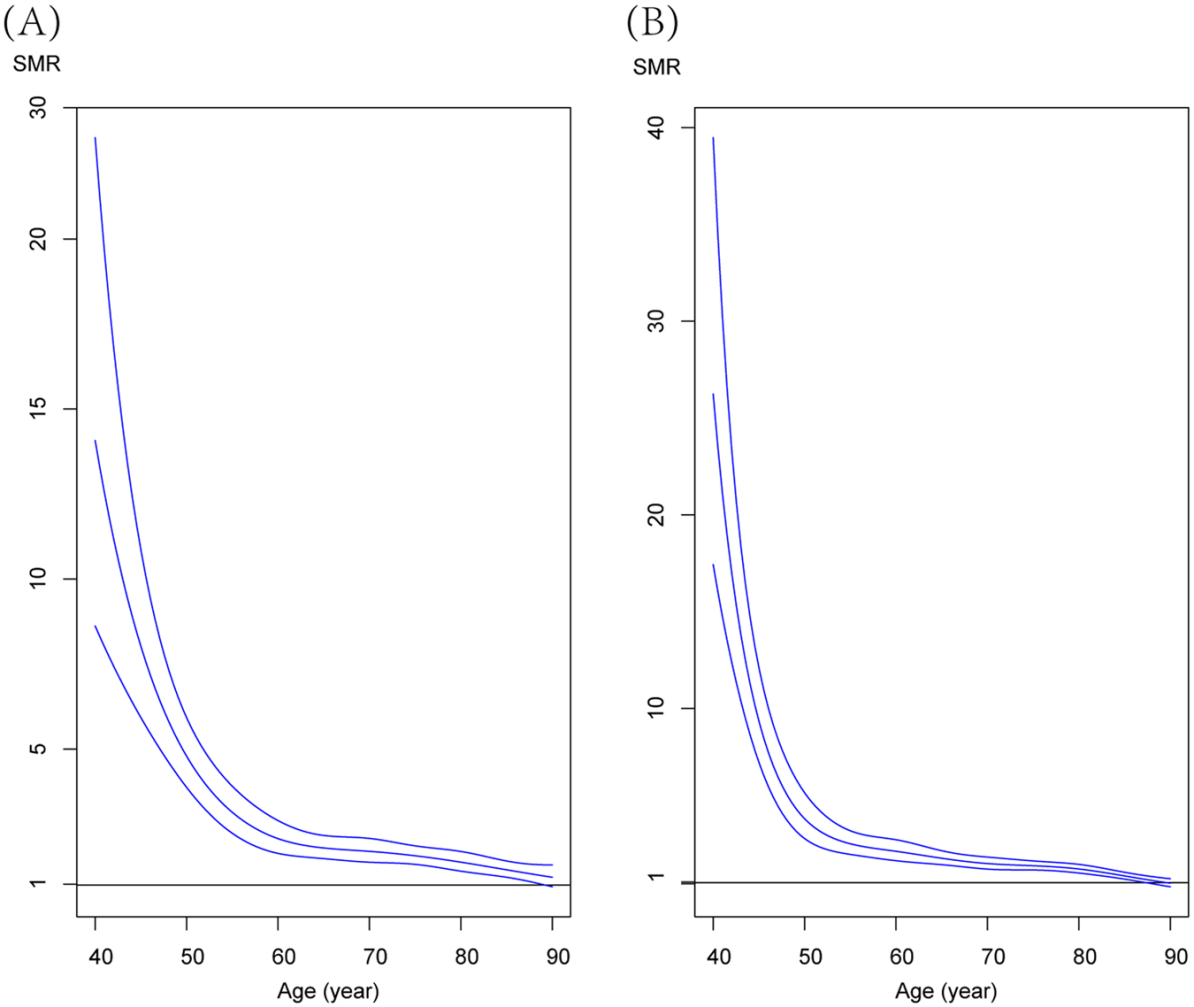
Idea of the splines is to smooth the SMR estimates and do inference from the curve figure, and continuous spline functions are fitted for age-group (<45, 46–55, 56–65, 66–75, 76–85, +85) among male (**A**) and female breast cancer (**B**) patients.

Nevertheless, we found that no significant difference in adjusted SMR between MBC and FBC ((SMR = 1.98, 95% CI = 1.83,2.14) for FBC and (SMR = 2.07, 95% CI = 1.88–2.28) for MBC). Similar trends of SMR by age were reveled, and SMR for both MBC and FBC was higher for young patients thereafter gradually declined (P < 0.001) (Fig. 5) (Table 3). Interestingly, SMR of MBC (SMR = 12.65, 95% CI = 6.57, 21.66) was significantly lower than that of FBC (SMR = 26.24, 95% CI = 16.92, 38.47) in young patients.

**Table 3:** Standardized mortality ratio (SMR) for age among matched male (N = 1,111) and female (N = 2,151) breast cancer patients.

Variables/ Age, year	Male Breast Cancer				Female Breast Cancer			
	Observed	Expected	Adjusted SMR*	P-Value	Observed	Expected	SMR*	P-Value
<45	11	1	12.65 (6.57, 21.66)	P < 0.001	23	2	26.24 (16.92, 38.47)	P < 0.001
45–55	51	10	5.13 (3.85, 6.67)		52	12	4.30 (3.23, 5.58)	
55–65	63	28	2.25 (1.74, 2.85)		91	35	2.62 (2.12, 3.20)	
65–75	123	61	2.01 (1.68, 2.39)		159	80	1.99 (1.69, 2.31)	
75–85	129	77	1.67 (1.40, 1.97)		224	131	1.71 (1.49, 1.94)	
>85	58	40	1.23 (0.94, 1.58)		86	88	0.97 (0.78, 1.20)	
**Total**	435	211	1.94 (1.76, 2.13)		635	348	1.83 (1.69–1.97)	

^*^Adjusted for year of diagnosis and race.

P for homogeneity of SMR between age groups.

#### Nomogram to predict 5-year or 10-year OS

**Figure 6:**
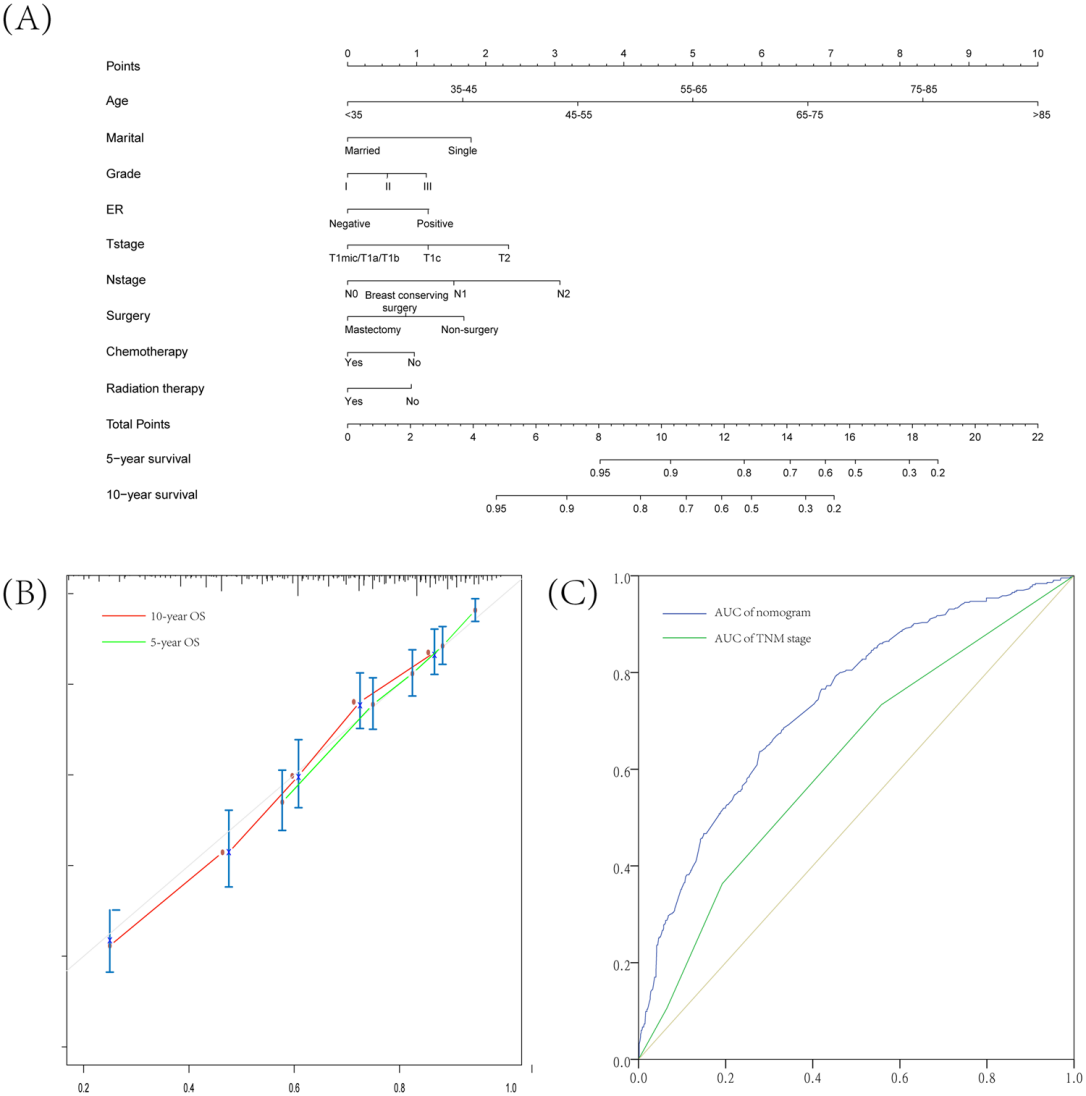
Nomogram and the internal calibration for predicting 5-year and 10-year overall survival of early male breast cancer patients. (**A**) Nomogram for predicting proportion of patients with 5-year and 10-year OS. (**B**) Plots depict the calibration of model in terms of agreements between predicted and observed 5-year and 10-year outcomes. Model performance is shown by the plot, relative to the 45-degree line, which represents perfect prediction. (**C**) ROC curves showed sensitivity and specificity of overall survival prediction by the TNM stage and risk score based on nomogram.

Associations of year of diagnosis, age, race/ethnic, marital status, histologic type, grade, ER and PR status, AJCC T stage, AJCC N stage, and systematic therapy data (i.e. surgery, chemotherapy and radiation therapy) with OS were analyzed based on the MBC cohort (Table 2), indicating that some of these clinicopathological variables are potential risk factors. Considering most of patients with MBC in this study had ductal carcinoma and were of white race, histological type and race were not included in nomogram. We constructed a prognostic nomogram including clinicopathological variables to predict 5-year or 10-year survival using training cohort (Fig. 6A), and validated the model internally (Fig. 6B). In internal validation, C-index for the nomograms to predict OS was 0.71 (95% CI, 0.66 to 0.76), whose calibration plots were presented in Fig. 6B. Compared with the TNM stage survival classification, the AUC of nomogram (0.74, 95% CI = 0.71 to 0.77) is higher than that of TNM stage (0.62, 95% CI = 0.58 to 0.68). These results consistently indicated that the predicting ability and discrimination of the models were generally good.

### Ethics approval

All procedures performed in studies involving human participants were in accordance with the ethical standards of institutional and/or national research committee and with the 1964 Helsinki declaration and its later amendments or comparable ethical standards.

## Discussion

In this large population-based cohort study, multivariate Cox proportional hazard model after matching men and women with breast cancer the latter group had a better prognosis. Nevertheless, this survival superiority did not remain among BC patients with age less than 40 years, lobular or ER-negative BC, and similar SMRs and its trends by age groups were observed between MBC and FBC except for young patients. To predict individual survival of MBC, a concise and clear nomogram was constructed, and the satisfactory performance from internal validation and AUC suggested that it is a valid tool.

In one of the largest comparative study conducted by Anderson *et al*.^6^, they revealed that the biology of MBC was resembled with late-onset FBC, and progress for men has lagged behind that for women. Despite the same registry with our study, comparability between groups is inadequate, and some modifier-effects were ignored (e.g. age, histological type and ER status). Recently, Iorfida *et al*.^7^ conducted a matched single-institution series, which matched all prognostic variables and made conclusion that men with breast cancer had a poorer disease-free survival and OS when compared with women. This study included 99 men and 198 women with breast cancer, but the power of sample size was not verified. On the contrast, in an age- and stage-matched cohort male and female breast cancer patients showed no significant difference in 5- and 10-year disease free survival and OS rates^20^. When neglecting imbalance of clinicopathological factors between male and female patients with breast cancer, most of prior comparative studies^21–23^ consistently found a statistically significant difference in favor of a better prognosis in FBC patients. After propensity score matching between MBC patients and FBC, our study unsurprisingly indicated that FBC patients had superior overall survival compared with MBC.

MBC tends to present at an older age, peak incidence was approximately at 71 years^24,25^. Age-frequency distribution for women was bimodal, whereas that of MBC was unimodal^26,27^. For absence of hormone periodic change like women, age is satisfying predictor for MBC prognosis, and current viewpoint treated it as post-menopausal BC^28^. In our subgroup analyses, we also found that the prognosis of MBC patients was no longer poorer than that of FBC in subset of patients less than 40 years. Actually, SMRs can better reveal the survival difference between MBC and FBC due to that men often have shorter life expectance and later peak incidence than women. Accordingly, a recent study^29^ showed similar SMR for the two genders, except for very young FBC, who had higher SMR, which were comparable with our results. It also means that MBC was resemble with old FBC, and old patients in general have low malignant breast cancers and they often die from co-morbidities. In addition of that, up to 74.2–93.7% of MBC were classified as ductal carcinomas compared with 67.4–83.6% of FBC^22,30^. Even though matched procedure yielded the balanced baseline characteristics, the highly skewed nature of the data set with 0.6% of MBC patients with lobular carcinoma (vs. 9.9% FBC patients with lobular carcinoma) probably contributed to the no difference in OS between lobular MBC and FBC.

The ER positive rates of MBC were more than 90%, which accounted for that invalid effect of this important biomarker on prognosis^22^. In our study, either comparative survival analyses matched with FBC, subgroup analyses, risk factor analyses or nomogram consistently indicated that ER-positive is a risk factor among MBC patients, for ER-negative MBC patients had better survival than ER-positive MBC and ER-negative FBC (Fig. 3B–D), respectively. Although these associations were marginally significance due to limited numbers of ER-negative MBC patients and ER negative is seldom among elderly male breast cancer patients, we also cannot ignore these identical results. A prior study identified significant differences in survival according to ER status among MBC patients^31^, showing that ER-positive patients acquired better prognoses than ER-negative MBC. In spite of these, potential bias was inferred that higher proportion (approximately 60%) of MBC with advanced/distant (III/IV) or unknown stage may lead to this result. In contrast, our survival comparison between ER-positive and ER-negative MBC patients had relatively comparable property in baseline data (Supplementary Table S1). We hypothesized that the limited efficacy of endocrine therapy for ER-positive patients with MBC can interpret this phenomenon. Although little randomized controlled trails tried to investigate most suitable endocrine therapy strategy for early ER-positive MBC patients, Eggemann *et al*. revealed that significantly increased OS in the group treated with tamoxifen compared with aromatase inhibitor group where 257 patients with MBC were treated with adjuvant endocrine therapy^32^. In addition, another retrospective study also showed that tamoxifen, as a standard care of adjuvant endocrine therapy, had satisfying efficacy and good tolerance^33^. These results warrant further exploration of potential benefits of other endocrine therapy for early-stage MBC in a controlled, prospective clinical trial setting.

As we all known, the most universal treatment for local disease is modified radical mastectomy for MBC, similarly, breast conserving surgery is accepted too^34^. Recent studies have shown that less invasive surgical approaches with no detectable decline in survival, and it added benefit of increased functional and psychological outcomes^35–39^, which was further validated by our study that different operation areas presented no significant differences in survival. Interestingly, interaction analyses revealed that more distinct survival difference between MBC and FBC was observed when surgical procedure changed from breast conserving surgery to mastectomy. It might be involved in that peculiar anatomy of the male mammary included less fat tissue, and the proposed ‘gas station’ effects of fat tissue on residual carcinoma cell will be cut^40^. Furthermore, we observed that the better prognosis was acquired when accepting radiation therapy and/or chemotherapy compared with those without adjuvant therapies, and consistent benefits were identified regardless of early or advanced BC^41,42^. Moreover, a recent study examined the effect of target-adjuvant drugs like mechanistic target of rapamycin (mTOR) inhibitors on subsets of MBC who was identified by transcriptomic investigation^43^, expectedly, and more practicable treatments will be utilized for MBC to improve prognosis.

Association of marital status and the prognosis of cancer such as hepatocellular carcinoma, pancreatic neuroendocrine tumor and tracheal cancer was widely explored before^44–46^, which consistently suggested that marital status is an independent prognostic factor. Single patients have the higher risk of overall death compared with married group in our study, which is comparable with prior studies^31,44–46^. Potential mechanisms of phenomenon are unknown, we supposed that widowed patients lack care contribute to poor prognosis of MBC.

The notable strength of this study is to compare the OS between patients with early FBC and MBC who were matched using propensity scores, and all that matters is that we identified the status of ER-positive as a potential risk factor for survival of MBC patients. To the best of our knowledge, we are also the first to explore nomograms to predict the individualized survival of early MBC patients, and this work can provide opportunities for clinicians to classify the patients according to risk scores, which can help select therapy strategies. Nevertheless, some limitations of our study should be acknowledged. Firstly, some therapy-associated data like endocrine therapy and targeted therapy is not available in the SEER database, which exert vital effects on prognosis of BC. Secondly, considering sample size and appreciate follow up, we gave up an important status of HER2, which is registered after 2010 in SEER database, and previous studies reported that a higher HER2 overexpression rate in MBC compared to FBC^9–11^. Lastly, we cannot control the quality of primary data, and pathological diagnosis from multiple hospital will lead to inevitable bias.

## Conclusion

In conclusion, significantly poorer OS among early MBC patients compared with FBC was found where the baseline characteristics by gender were well balanced, higher proportion of ER positivity of early MBC patients probably contributes to this poorer prognosis. Similar SMRs and its trends by age groups were observed between MBC and FBC except for young patients (<45 years old) whose SMR was higher in FBC than corresponding male patients. The nomogram was developed for clinicians to predict 5-year and 10-year individualized survival of early MBC, whose better performances than TNM stage classification suggested that it is a valid tool.
